# HBx promotes hepatocellular carcinoma progression by repressing the transcription level of miR-187-5p

**DOI:** 10.18632/aging.204921

**Published:** 2023-08-01

**Authors:** Yang Deng, La Wang, Yingjie Zhang, Dandan Sun, Hang Min, Hao Zhou, Chengchen Xu, Na Xu, Fengwu Qiu, Jingjiao Zhou, Jun Zhou

**Affiliations:** 1 College of Life Sciences and Health, Wuhan University of Science and Technology, Wuhan 430081, China; 2 Hubei Institute of Blood Transfusion, Wuhan Blood Center, Wuhan 430033, China

**Keywords:** hepatocellular carcinoma, HBx, miR-187-5p, E2F1

## Abstract

HBV-associated hepatitis B virus x protein (HBx) plays multiple roles in the development of hepatocellular carcinoma. In our prior study, we discovered that miR-187-5p expression was inhibited by HBx. To investigate the underlying molecular mechanism of HBx-mediated miR-187-5p downregulation in hepatocellular carcinoma cells, effects of HBx and miR-187-5p on hepatoma carcinoma cell were observed, as well as their interactions. Through *in vitro* and *in vivo* experiments, we demonstrated that overexpression of miR-187-5p inhibited proliferation, migration, and invasion. Simultaneously, we observed a dysregulation in the expression of miR-187-5p in liver cancer cell lines, which may be attributed to transcriptional inhibition through the E2F1/FoxP3 axis. Additionally, we noted that HBx protein is capable of enhancing the expression of E2F1, a transcription factor that promotes the expression of FoxP3. In conclusion, our results suggest that the inhibitory effect of HBx on miR-187-5p is mediated through the E2F1/FoxP3 axis. As shown in this work, HBx promotes hepatoma carcinoma cell proliferation, migration, and invasion through the E2F1/FoxP3/miR-187 axis. It provides a theoretical basis for finding therapeutic targets that will help clinic treatment for HCC.

## INTRODUCTION

Hepatocellular carcinoma (HCC) is the sixth of the most common cancers and the fourth leading cause of the worldwide cancer death after lung, colorectal and gastric cancers [1]. Hepatitis B virus (HBV) infection leading to HCC is a worldwide health issue. Despite the rapid development of diagnostic techniques and treatments for HBV-related HCC (HBV-HCC), the survival rate of HCC and HBV-infected individuals has not improved. The HBV x protein (HBx) encoded by the HBV genome is hypothesized to have a role in hepatocarcinogenesis. Mounting evidence shows that HBx increases HCC growth through a variety of mechanisms, including gene expression changes, protein breakdown disruption, signaling pathway modification, and non-coding RNA dysregulation. The mechanisms for these biological processes are not completely understood [2–4].

MicroRNAs (miRNAs) are short non-coding RNAs of 19–25 base pairs that control gene expression by binding to mRNA targets’ 3′-untranslated region (3′-UTR) [5]. It is predicted that the human genome can encode more than 2600 miRNAs, and a single miRNA can target and regulate the expression of at least 200 mRNAs and regulate the expression of more than 15,000 genes [6]. miRNA can regulate various carcinogenic molecules, signaling pathways, and function as unstable regions of the genome [7]. HBx is reported to change the miRNA expression profile in liver cancer cells, which can be used as potential diagnostic markers for HCC. Recent studies indicated that the miR-187-5p gene on chromosome 18q12.2 played a significant role in tumor regulation [8, 9]. miR-187-5p has been shown to be down-regulated in giant cell tumors of bone [8], and non-small cell lung cancer [9] but up-regulated in triple negative breast cancer [10], gastric cancer [11], and bladder cancer [12] as an oncogene.

FoxP3 is one of the fork-head box (fox) transcription factor and functions as a transcription factor in multiple cells [13]. The multifaceted nature of Foxp3, a transcription factor of considerable importance, cannot be overstated. Not only does it play a crucial role in promoting transcription, but it also exhibits suppressive properties. Intriguingly, studies have shown that high expression of Foxp3 can activate T cells, adding yet another layer of complexity to its already impressive repertoire of functions [14]. It has been reported that FoxP3 is expressed both in CD4+ T regulatory cells and tumor cells, including pancreatic cancer cells, melanoma cells etc. [15–17]. Studies have shown that mutations in the FoxP3 gene are widely involved in human autoimmune diseases [18]. Furthermore, TGF-2 and IL-10 are among the cytokines FoxP3 regulates, and thus promotes tumor cells to evade immunity and to deteriorate further [19]. E2F1 belongs to the E2Fs family which is a transcription factor of 437 amino acids. It contains a DNA binding domain (DBD) and a trans-activation domain (TDD), necessary for binding to target genes [20]. Some amino acid sequence in the TDD bound to retinoblastoma protein (pRB) inhibits the transcriptional activity of E2F1 [21] in turn. Moreover, E2F1 was found to promote gene transcription [22] by interacting with transcription factors in members of the DP family to form an E2F1/DP heterodimer structure. By co-factor binding and subcellular localization [23]. E2F transcriptional activity is regulated by transcriptional and translational processes, post-translational changes, and protein degradation throughout the cell cycle. Based on bioinformatic research, we discovered that HBx inhibits miR-187-5p expression in HCC cell lines [24]. The association between HBx and miR-187-5p and the mechanism underneath triggered our interest. In this study, we explored the interaction mechanism among HBx, miR-187-5p and transcription factors E2F1 and FoxP3 in hepatocellular carcinoma cells. In order to clarify the molecular mechanism involved in HCC occurrence and progression by HBx.

## MATERIALS AND METHODS

### Cell and cell culture

The normal liver epithelial cell line LO2, as well as the HCC cell lines Huh-7 and HepG2 were provided by Wuhan Warner Biotechnology Co., Ltd., China. Dulbecco’s modified Eagle’s medium (Meilun, Dalian, China) were used to culture Huh-7 and HepG2 cells, and Roswell Park Memorial Institute 1640 medium (Meilun, Dalian, China) was used to culture LO2 cells, both of these needed to contained 10% fetal bovine serum (Every Green, Hangzhou, China) and 1% Penicillin/chloramphenicol (Meilun, Dalian, China) and then was incubated at 37°C with 5% CO_2_.

### Plasmid construction

cDNAs of HBx, FoxP3 and E2F1 were amplified and sub-cloned into pcDNA3.1-Flag (Invitrogen, Carlsbad, CA, USA). 3′-UTR fragment of wild-type (WT) CDH2 and mutant (MUT) CDH2 were cloned into the dual-luciferase expression vector pmirGLO (Invitrogen, Carlsbad, CA, USA). The promoter region of WT and MUT of miR-187-5p (WT-miR-187-5p-Luc and MUT-miR-187-5p-Luc), WT and MUT of FoxP3 (WT-FoxP3-Luc and MUT-FoxP3-Luc) were cloned into the luciferase vector pGL3-Promoter (Addgene, Watertown, MA, USA). All cloning primers have been shown in Supplementary Table 1.

### Plasmid transfection

In all cases, constructed plasmid vectors have been isolated using the CWBiotech endofree plasmid midi kit (Beijing, China). Transfection of the plasmid vectors was achieved with Lipofectamine 2000 (Invitrogen, Carlsbad, CA, USA) following manufacturer’s instructions.

### Quantitative real-time PCR (qRT-PCR) assay

TRIzol reagent was used to separate cellular RNA from hepatoma carcinoma cell according to the manufacturer’s instructions (Invitrogen, Carlsbad, CA, USA). The reverse transcription procedure, which was required to synthesize single-strand complementary DNA (cDNA), was carried out using a reverse transcription kit (Vazyme, Nanjing, China). The Hairpin-it miRNA real-time quantitative reverse transcription polymerase chain reaction (qRT-PCR) kit was used to reverse transcribe miR-187-5p (Vazyme, Nanjing, China). Following that, qRT-PCR was performed on the Bio-Rad CFX96 Real-Time PCR Detection Machine (Bio-Rad Laboratories, Hercules, CA, USA) using 2*SYBR Green qPCR Master Mix (Yeason, Shanghai, China). U6 functioned as internal reference of miR-187-5p. GAPDH functioned as internal reference of FoxP3 and E2F1. In order to measure relative gene expression, the 2−ΔΔCt method was applied using primers synthesized by TSINGKE (Beijing, China). All primers are shown in Supplementary Table 2.

### Western blot

Strong RIPA lysis buffers (Meilun, Dalian, China) were used to lye the cells 48 hours after transfection, and total protein was recovered by high-speed centrifugation. The Enhanced BCA Protein Assay Kit (Meilun, Dalian, China) was used to quantify total protein, and 30 g of total protein was segregated using sulfate-polyacrylamide gel electrophoresis (SDS-PAGE). The entire proteins were then isolated and put into polyvinylidene fluoride (PVDF). Close for 1 hour at room temperature. The primary antibodies were incubated overnight at 4°C. The following primary antibodies were used: anti-GAPDH antibody (AC002; Abclonal, Wuhan, China; 1:5000 dilution), anti-FoxP3 antibody (ab22510, Abcam; 1:1000 dilution), anti-E2F1 antibody (A2067; Abclonal; 1:1000 dilution), anti-Flag antibody (20543-1-AP; Proteinech, Wuhan, China; 1:1000 dilution), anti-CDH2 antibody (A19083; Abclonal; 1:1000 dilution), anti-PCNA antibody (A12427; Abclonal; 1:1000 dilution), and anti-Snail antibody (A11794; Abclonal; 1:1000 dilution). A goat anti-mouse IgG(H+L) secondary antibody conjugated with horseradish peroxidase (HRP) was then added (31432; Invitrogen, Carlsbad, CA, USA; 1:2000 dilution) to the membranes, followed by washing with wash buffer and incubation with a goat anti-mouse or a goat anti-rabbit IgG(H+L) secondary antibody.

### Luciferase reporter assay

For the luciferase reporter assay, 1 × 10^5^ cells per well were pre-incubated overnight in a 24-well plate before being co-transfected with 100 ng of luciferase vectors and 400 ng of protein expression plasmids of FoxP3 and E2F1, 50 mol/L miR-187-5p or FoxP3 mimic and their relative control plasmids. The fluorescence intensity of 293T cells 24 hours after transfection was measured using a dual luciferase reporter assay (Promega, Madison, MA, USA).

### 5-Ethynyl-2′-deoxyuridine (EdU)-labeling assay

The 5-ethynyl-2′-deoxyuridine (EdU)-labeling procedure was similar as Li’s essay [25]. EdU was a thymidine nucleoside analogue that could be integrated into newly synthesized DNA and utilized to identify growing cells. Huh-7 cells were incubated in a 24-well plate for overnight, with a density of 4 × 10^4^ cells per well. The Cell-Light^™^ EdU *In vitro* Cell Proliferation Imaging Kit was used for transfection. EdU-Labeling Assay was conducted according to the manufacturer’s instructions (Ribobio, Guangzhou, China). After counterstaining the cultivated cells with 4, 6-diamidino-2-phenylindole, pictures of the cells were taken using a confocal laser scanning microscope (OLYMPUS, Japan). The cells were further examined by dividing the total number of cells by the number of EdU+ cells. Every experiment was carried out in triplicate.

### Cell counting kit-8 (CCK-8) assay

Incubation was performed overnight with 1000 Huh-7 cells per well of 96-wells in DMEM. Cells were counted at various time points by measuring OD450 nm with a Spectra Max i3 system (Molecular Devices) as directed by the manufacturer. All experimental results were stripped of the value of the blank control. For each experiment, the cell numbers in three images were averaged. All experiments were performed three times.

### Wound-healing assay

6-well plates were seeded with Huh-7 cells for wound-healing experiments. Wounding was induced by scratching the monolayer with a 200 μL micropipette tip 24 or 48 hours after transfection. The cells were then placed in a 5 percent CO2 incubator chamber at 37°C. Using a microscope, images were taken after 24 or 48 hours. For each experiment, the cell numbers in three images were averaged. All experiments were performed three times.

### Migration assay

The motility of cells was evaluated in Trans-well chambers (Corning, NY, USA) with 8-m-pore polycarbonate membranes essentially as described in Li’s [26]. A total of 40000 cells were reconstituted in DMEM without FBS and put into the top chamber of the Transwell chamber. DMEM with 10% FBS was introduced to the bottom compartment. After 24 hours, cells were fixed in 4 percent paraformaldehyde, stained with Giemsa. The cells which did not migrate were scraped, photographed by inverted microscope (Olympus, Japan). All experiments were repeated for three times. For each experiment, three fields of each well were chosen and averaged for migrated cell counting.

### Nude mouse xenograft model

Female 4-week-old BALB/c nude mice were divided into two groups randomly. Mice for normal control group were injected with pLKO.1-Huh-7 cells. Mice for over expression of miR-187 were injected with pLKO.1-miR-187-Huh-7 cells. 0.2 mL of cell suspension that containing 2 × 10^7^ cells was injected into the left flank of each mouse. A digital caliper was used to measure tumor diameters once every week. The mice were euthanized and photographed after 28 days. Using a digital caliper, tumor tissues were taken out, photographed and weighed. Immunohistochemical staining was finished by Wuhan Servicebio Technology Co., Ltd., China

### Bioinformatic analysis

The bioinformatic analysis websites UALCAN, TargetScan, ALGGEN, and JASPAR were used to determine miR-187-5p expression, the miR-187-5p binding site to CDH2, the promoter binding site of E2F1 to FoxP3, the promoter binding site of FoxP3 to miR-187-5p, and the classical binding sites of E2F1 or FoxP3.

### Statistical analysis

To determine statistical significance, the student *t*-test or one-way analysis of variance (ANOVA) were utilized. ^*^P < 0.05 was regarded as statistically significant.

### Availability of data and materials

All data generated or analyzed during this study are included in this published article.

## RESULTS

### HBx promoted proliferation, migration and invasion of hepatoma carcinoma cells

**Figure 1:**
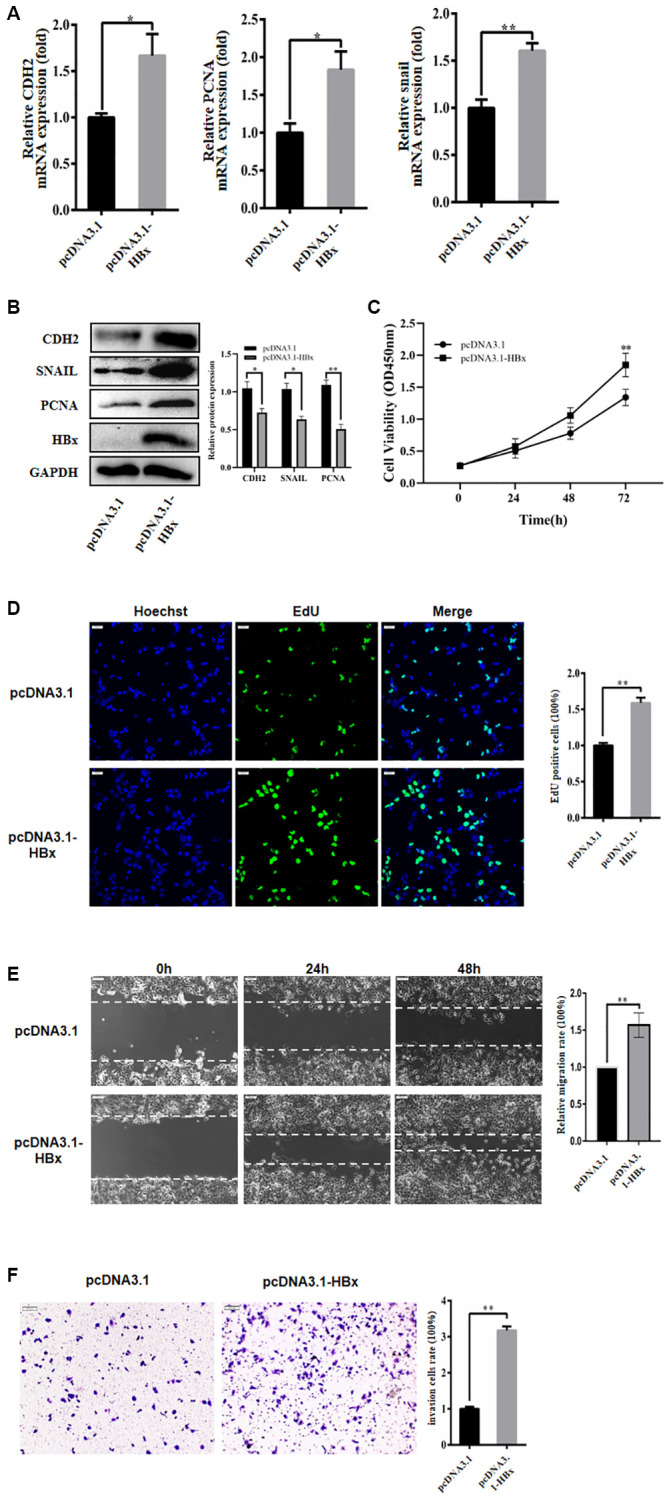
**HBx promoted proliferation, migration and invasion of hepatoma cells.** (**A**) HBx overexpression promoted the expression of PCNA, CDH2, and snail by RT-qPCR detection. (**B**) HBx elevated the expression of proliferative migration markers by western blotting. (**C**, **D**) EdU and CCK-8 assays confirmed the effect of HBx overexpression on the proliferation of Huh-7 cells. (**E**, **F**) HBx overexpression also promoted migration and invasion by both wound-healing assays and Transwell. The experiments were repeated at least 3 independent times. (**C**–**F**) Data was presented as mean ± SD from three independent experiments. ^*^p < 0.05; ^**^p < 0.01.

In this study, we investigated the impact of HBx on the proliferation, migration, and invasion of HCC cells through the evaluation of molecular markers’ expression after overexpressing HBx in Huh-7 cells. We examined the mRNA expression of respective markers by qRT-PCR and found that the expression of N-cadherin and Snail was greatly increased after HBx overexpression (Figure 1A, 1B). Subsequently, we assessed cell proliferation using both EdU and CCK-8 assays in the Huh-7 cell line. Our results showed that HBx overexpression accelerated the cell cycle in the CCK-8 assay and also promoted Huh-7 cell growth in the EdU experiment (Figure 1C, 1D). At the same time, we found that overexpression of HBx promoted migration and invasion of hepatocellular carcinoma cell line (Figure 1E, 1F). These findings suggest that HBx may play a role in promoting the proliferation, migration, and invasion of hepatoma carcinoma cells.

### HBx inhibited the expression of miR-187-5p

**Figure 2:**
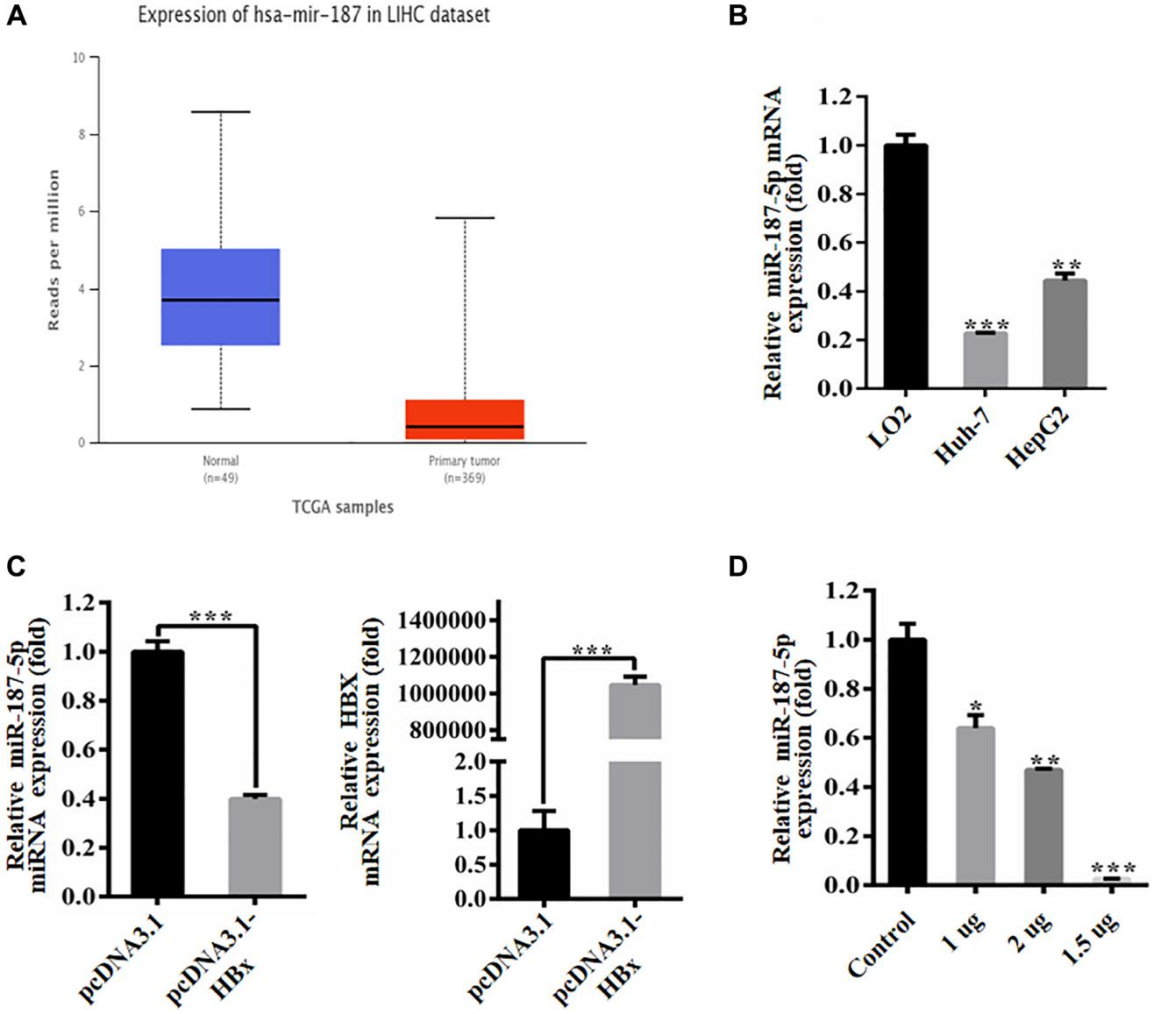
**HBx inhibited the expression of miR-187-5p.** (**A**) Decreased expression of miR-187-5p in hepatocellular carcinoma identified by UALCAN database. (**B**) Decreased expression of miR-187-5p by RT-qPCR detection in Huh-7 and HepG2 cells. (**C**) HBx overexpression significantly reduced miR-187-5p expression by RT-qPCR. (**D**) The expression of miR-187-5p decreased with the increase of HBx expression. The experiments were repeated at least 3 independent times. ^*^p < 0.05; ^**^p < 0.01; ^***^p < 0.001.

This study aimed to investigate the potential impact of HBx on the role of miR-187-5p in liver cancer cells. We leveraged the TCGA database to obtain expression data of miR-187-5p from hepatoma tissues of HCC patients, which revealed a significant reduction in miR-187-5p expression in hepatoma tissues compared to normal tissues (Figure 2A). Further qRT-PCR experiments were conducted to verify the expression level of miR-187-5p in human normal liver cells (LO2) and human hepatoma carcinoma cells (Huh-7 and HepG2), where we observed a substantial decrease in miR-187-5p expression in hepatoma carcinoma cells compared to human normal liver cells (Figure 2B).

We also explored the relationship between HBx and miR-187-5p. To this end, we transfected Huh-7 cells with pcDNA3.1 and pcDNA3.1-HBx for 48 hours and assessed the expression of miR-187-5p using qRT-PCR (Figure 2C). Our findings demonstrated a dose-dependent reduction in miR-187-5p expression when the pcDNA3.1-HBx plasmid was translocated into the Huh-7 cell line (Figure 2D). Specifically, the inhibitory effect of HBx on miR-187-5p was most pronounced at a transfection dose of 2 μg. These observations provide insights into the potential interactions between HBx and miR-187-5p, which may have implications for HCC progression.

### miR-187-5p inhibited proliferation, migration and invasion of hepatoma carcinoma cells

**Figure 3:**
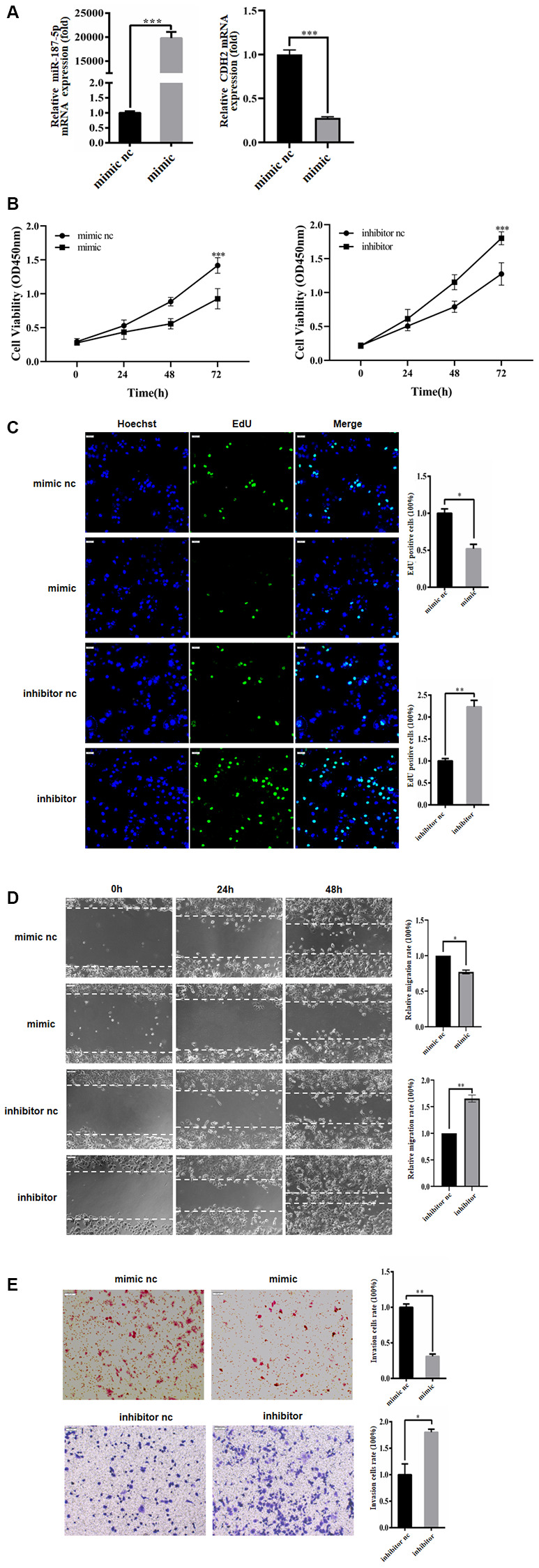
**miR-187-5p inhibited proliferation, migration and invasion of hepatoma cells.** (**A**) The transfection efficiency of miR-187-5p mimics and inhibitors was tested by RT-qPCR. (**B**, **C**) miR-187-5p mimics inhibited cell proliferation while miR-187-5p promoted cell proliferation by CCK-8 assays and EdU assays. (**D**, **E**) The effects of miR-187-5p mimics and inhibitors on migration and invasion of Huh-7 cell by Wound-healing and Transwell assays. The experiments were repeated at least 3 independent times. (**B**–**E**) Data was presented as mean ± SD from three independent experiments. ^*^p < 0.05; ^**^p < 0.01; ^***^p < 0.001.

To investigate the influence of miR-187-5p on hepatoma carcinoma cells, Huh-7 cells were transfected with either miR-187-5p mimics or negative control, followed by qRT-PCR to evaluate CDH2 mRNA expression. The results revealed that transfection with miR-187-5p mimics led to a noticeable reduction in CDH2 expression in Huh-7 cells, indicating a decrease in cell proliferation and cell cycle progression as demonstrated by EdU and CCK-8 assays (refer to Figure 3A–3C). Subsequently, wound healing assays were conducted using 6-well plates after transfecting the Huh-7 cells with miR-187-5p mimics or inhibitors for 48 hours. The findings indicated that the transfection of miR-187-5p mimics led to a significant decrease in Huh-7 cell migration ability compared to the control group. On the other hand, miR-187-5p inhibition transfection led to enhanced cell migration ability, suggesting that miR-187-5p may have a suppressive effect on Huh-7 cell migration (refer to Figure 3D). To further explore the impact of miR-187-5p on the invasive capacity of hepatoma carcinoma cells, Transwell assays were performed. Intriguingly, the invasive ability of Huh-7 cells with miR-187-5p mimics expression was significantly reduced relative to the control group. Conversely, the Huh-7 cells with miR-187-5p suppressor expression exhibited a considerable increase in invasive ability. In light of these observations, it can be deduced that miR-187-5p may modulate the invasive ability of liver cancer cells (refer to Figure 3E).

### miR-187 inhibited HCC growth *in vivo*

**Figure 4:**
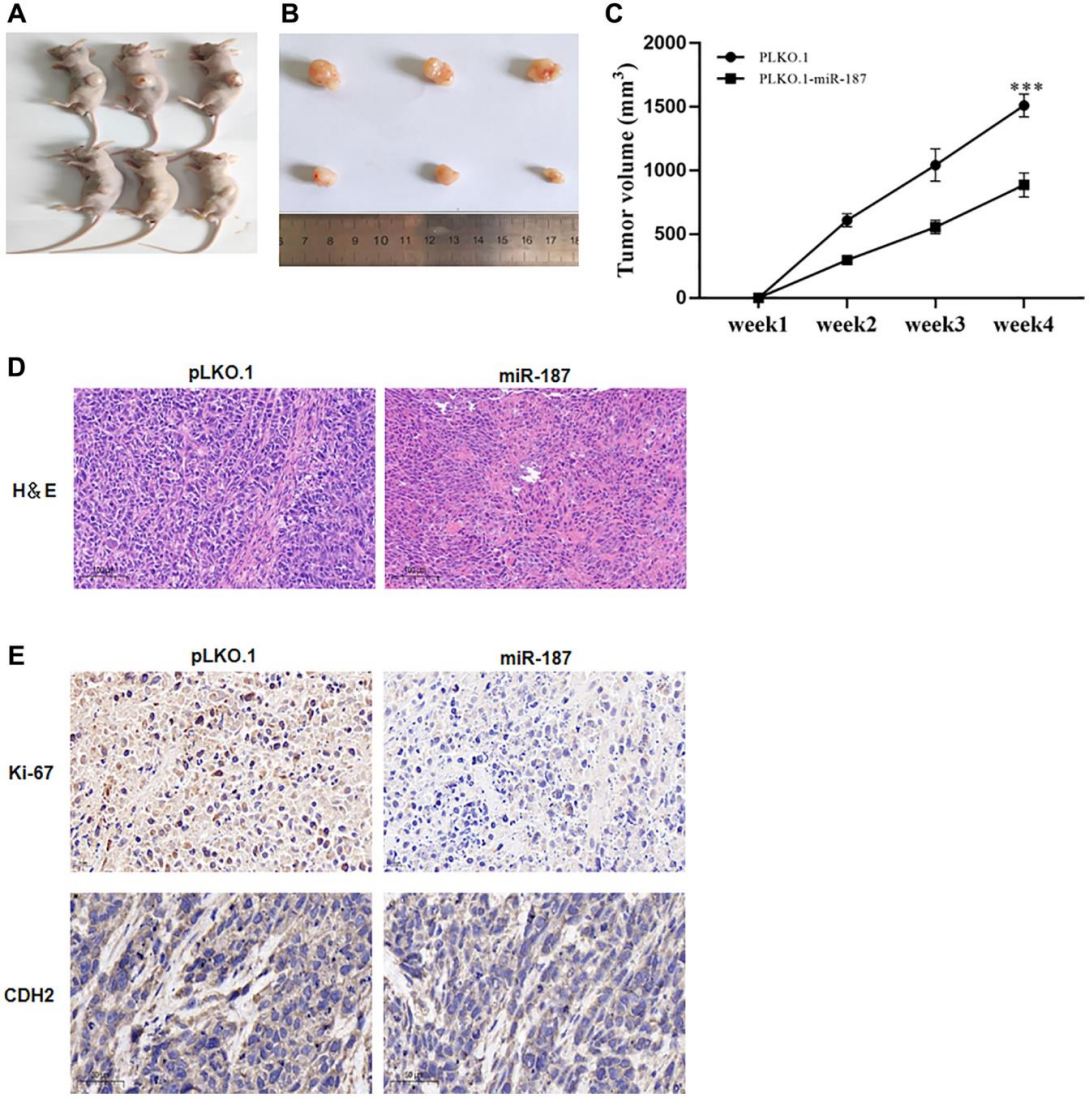
**miR-187-5p inhibited proliferation, migration and invasion of hepatoma cells.** (**A**–**C**) miR-187-5p overexpression resulted in a blunted tumor growth in terms of tumor weight and volume. (**D**) The engrafted tumors showed by H&E staining. (**E**) IHC analysis of Ki-67 and CDH2 protein expression.

*In vivo* experiments have confirmed the impact of miR-187 on tumor development. Specifically, nude mice were injected with Huh-7 cells that had been transduced with lentivirus expressing pLKO, pLKO.1-NC, and pLKO.1-miR-187, respectively. The resulting tumor growth was then observed and compared between the two groups. As depicted in Figure 4A, 4B, overexpression of miR-187-5p led to a suppressed tumor growth, as evidenced by reduced tumor weight and size, whereas the control group exhibited accelerated tumor growth (Figure 4C). Subsequently, subcutaneously transplanted tumors were analyzed via H&E staining, which showed that miR-187-5p overexpression led to a decrease in the degree of pathological lesions within the tumors (Figure 4D). Notably, compared to the NC group, a significant reduction in nuclear size and lighter nuclear staining were observed in the miR-187 overexpression group. Immunohistochemistry was then performed to measure CDH2 and Ki67 expression levels within the tumors. Results revealed that elevated levels of CDH2 and Ki67 expression were detected in the control group (Figure 4E), while down-regulated levels were observed in the miR-187 overexpression group, further supporting the suppressive effect of miR-187 on tumor growth.

### miR-187-5p bound to 3′-UTR of CDH2

**Figure 5:**
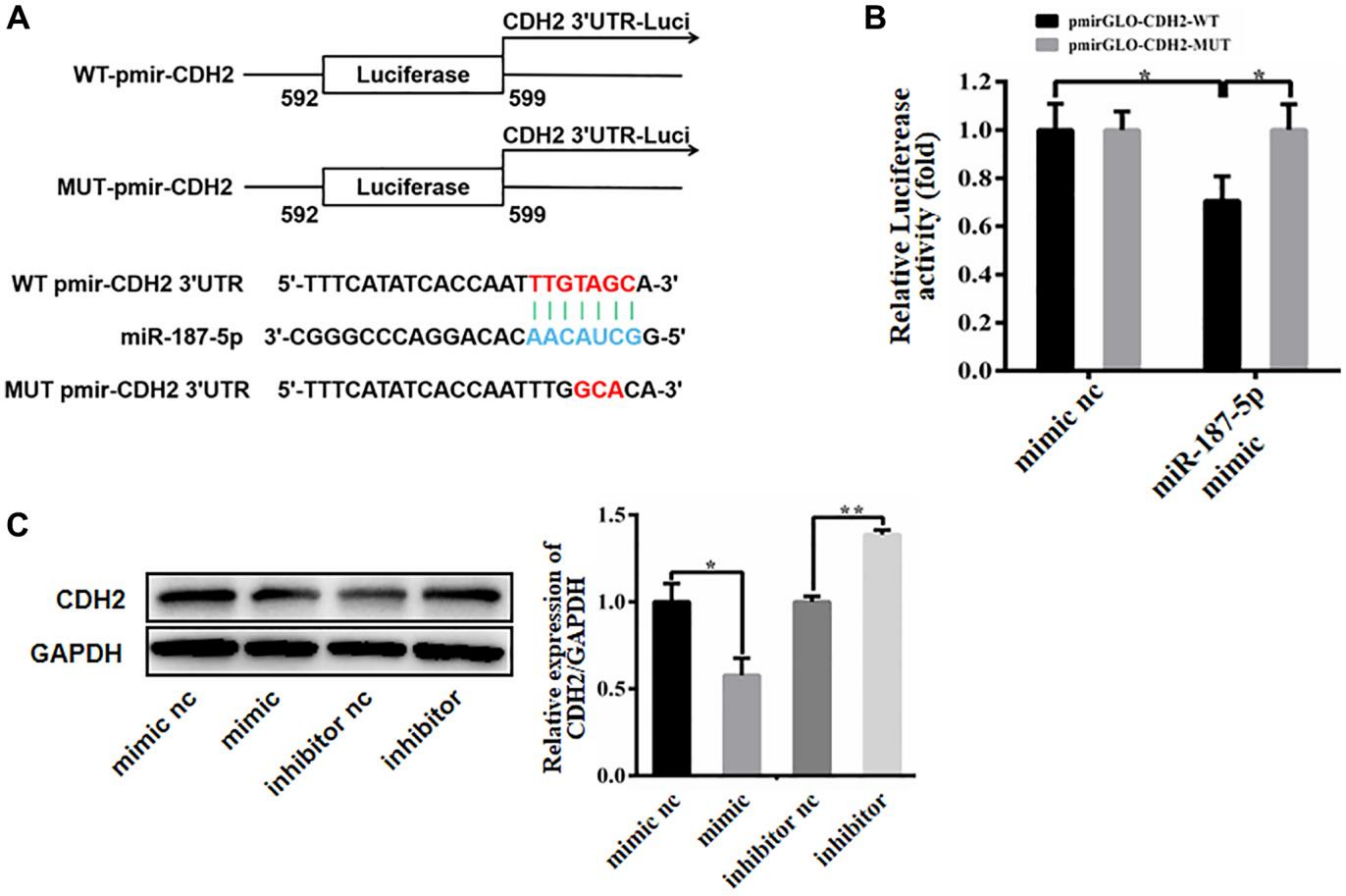
**miR-187-5p bound to 3’-UTR of CDH2.** (**A**) The putative seed recognition site in the 3′-UTR of CDH2 for miR-187-5p. (**B**) Luciferase reporter assay in HEK-293T cells. miR-187-5p significantly inhibited the luciferase activity of vector carrying 3′-UTR of CDH2, compared with control vector. (**C**) Regulation of CDH2 expression by miR-187-5p with western blotting. The experiments were repeated at least 3 independent times. ^*^P < 0.05; ^**^P < 0.01.

We predicted the 3′-UTR of CDH2 using the TargetScanHuman website tool to investigate how miR-187-5p regulates CDH2 expression. The result discovered a miR-187-5p-specific binding site (Supplementary Figure 1A). To determine whether miR-187-5p binds to the CDH2 3′-UTR, two luciferase vectors containing either the WT-CDH2-3′-UTR (WT-pmirGLO-CDH2) or the MUT-CDH2-3′-UTR (MUT-pmirGLO-CDH2) were generated (Figure 5A). When WT-pmirGLO-CDH2/MUT-pmirGLO-CDH2 and OE-miR-187-5p/OE-Ctrl were co-transfected into 293T cells. Overexpression of miR-187-5p was shown to inhibit the activity of the CDH2 WT luciferase vector and had lightly effect on the activity of the MUT vector (Figure 5B). MiR-187-5p overexpression may reduce CDH2 protein levels, whilst miR-187-5p inhibition might increase CDH2 protein levels (Figure 5C). All of the findings showed that miR-187-5p decreased CDH2 expression by targeting its 3′-UTR.

### FoxP3 bound to promoter of miR-187-5p

**Figure 6:**
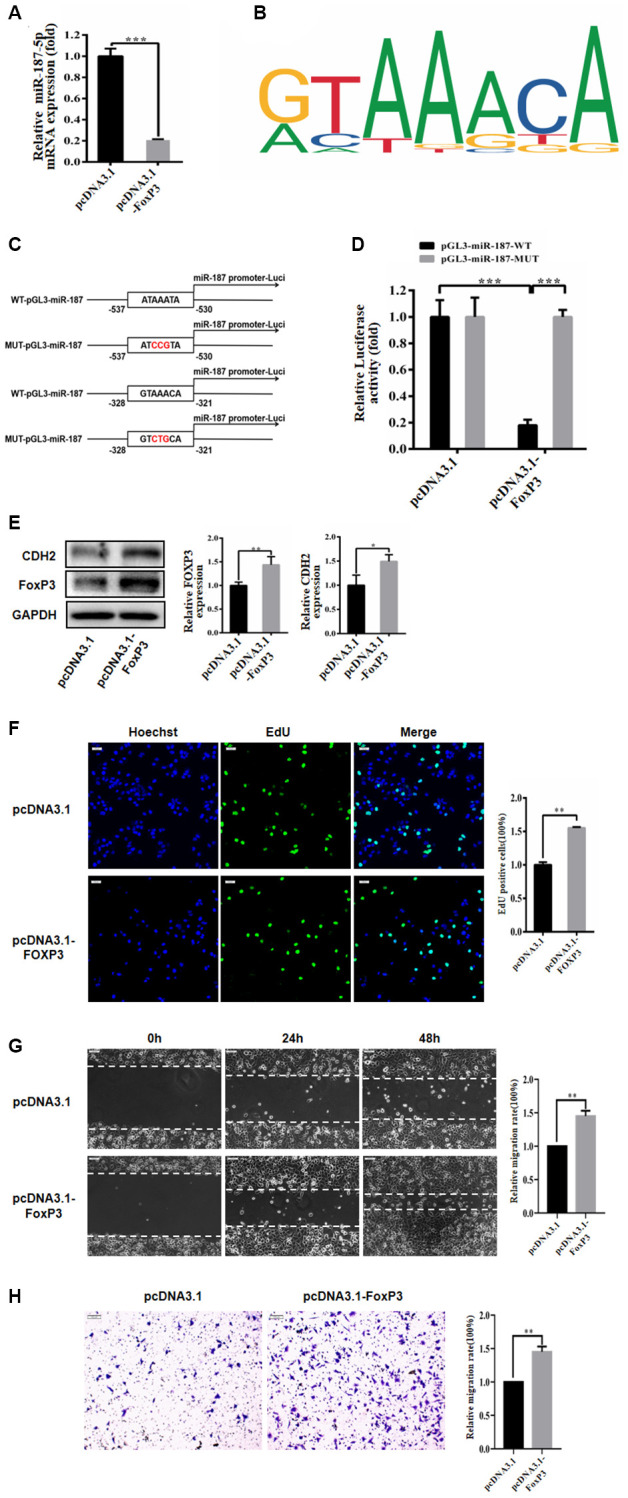
**FoxP3 bound to promoter of miR-187-5p.** (**A**) FoxP3 overexpression significantly decreased the expression level of miR-187-5p by RT-qPCR. (**B**) The FoxP3 binding site in the miR-187-5p promoter region. (**C**, **D**) Luciferase reporter assay in HEK-293T cells showed miR-187-5p significantly inhibited the luciferase activity of the vector carrying 3′-UTR of CDH2, compared with control vector. (**E**) FoxP3 significantly promoted the expression of CDH2 by western blotting. (**F**) Overexpression of FoxP3 promoted the proliferation of Huh-7 cells through EdU assays. (**G**) Overexpression FoxP3 increased the rate of wound closure. (**H**) In Transwell assays, results demonstrated more invasion number of hepatoma cells transfected with FoxP3, compared with control. The experiments were repeated at least 3 independent times. (**F**–**H**) Data was presented as mean ± SD from three independent experiments. ^*^P < 0.05; ^**^P < 0.01; ^***^p < 0.001.

We utilized the PROMO website to predict the promoter and determined that FoxP3 can bind to the promoter region of miR-187-5p (Supplementary Figure 1B). Subsequently, we transfected Huh-7 cells with pcDNA3.1 and pcDNA3.1-FoxP3, and our qRT-PCR analysis showed that overexpression of FoxP3 caused a significant reduction in miR-187-5p expression (Figure 6A). Moreover, we identified a binding site for FoxP3 within the miR-187-5p promoter region (Figure 6B). To further investigate the regulatory effect of FoxP3 on miR-187 expression, we constructed luciferase reporter plasmids with wild-type (WT pGL3-miR-187-5p) and mutant (MUT pGL3-miR-187-5p) miR-187 promoter sequences (Figure 6C), which were co-transfected into 293T cells with pcDNA3.1 and pcDNA3.1-FoxP3. The luciferase activity assay demonstrated that overexpression of FoxP3 significantly reduced the luciferase activity of the WT pGL3-miR-187-5p vector but had only a minimal effect on the MUT pGL3-miR-187-5p vector (Figure 6D).

Furthermore, we investigated the impact of FoxP3 on CDH2 expression in the Huh-7 cell line using Western blot analysis, which revealed that FoxP3 markedly increased CDH2 protein expression compared to the control group (Figure 6E). Subsequently, we evaluated the effect of FoxP3 on the proliferation, migration, and invasion of hepatoma carcinoma cells by transfecting Huh-7 cells with pcDNA3.1 and pcDNA3.1-FoxP3 for 72 hours. Our results demonstrated that overexpression of FoxP3 significantly enhanced the proliferation of Huh-7 cells, as assessed by the EdU assay (Figure 6F). Moreover, wound healing and Transwell experiments revealed that overexpression of FoxP3 increased the closure rate of the wound and the invasion ability of Huh-7 cells compared to the control group (Huh-7 cells transfected with pcDNA3.1) (Figure 6G, 6H), indicating that FoxP3 promotes migration and invasion of hepatoma carcinoma cells.

### HBx Elevated FoxP3 Expression by Interaction with E2F1

**Figure 7:**
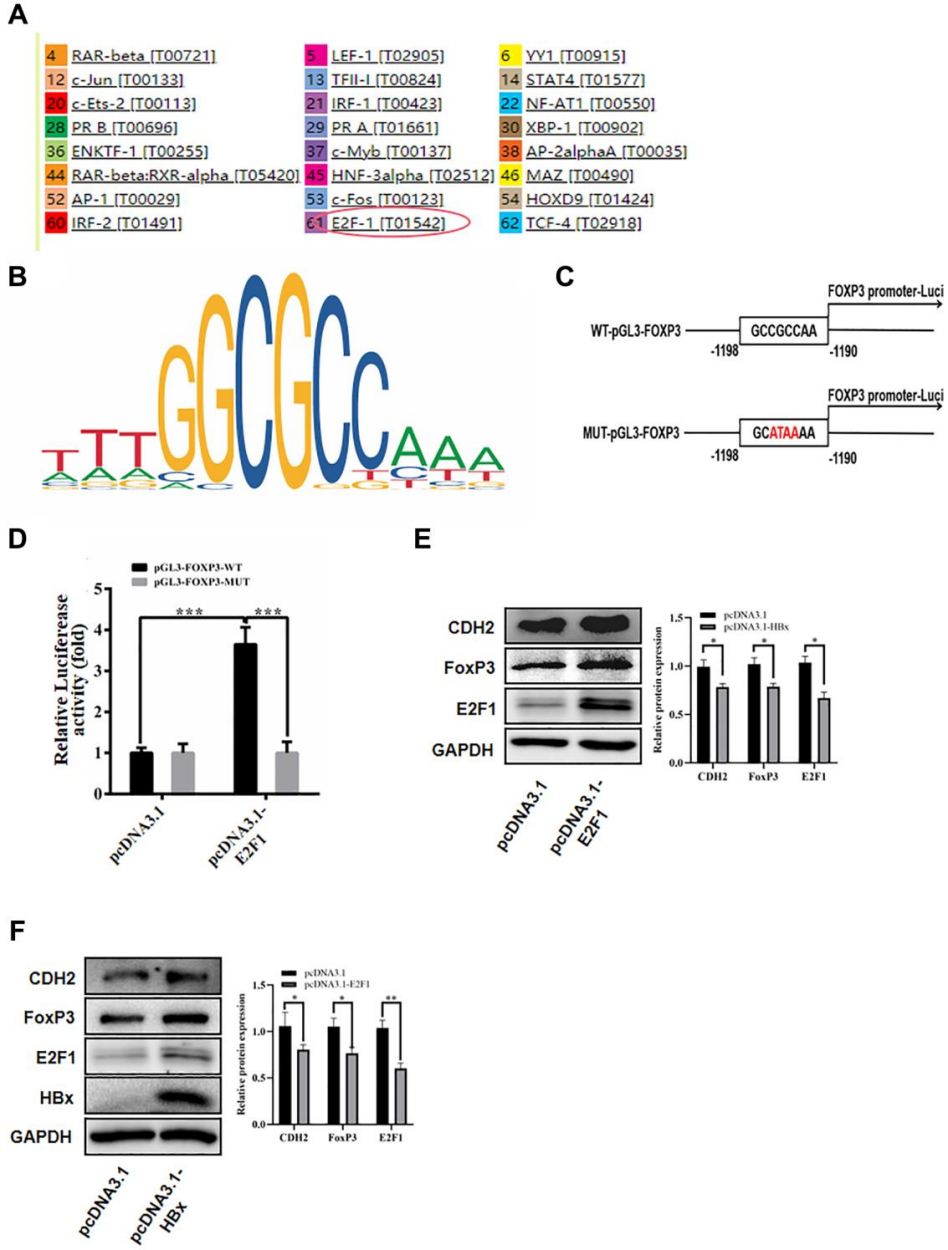
**HBx promoted FoxP3 expression by E2F1.** (**A**) Used PROMO website to predict the promoter of FoxP3. (**B**) E2F1 binding site in the FoxP3 promoter region. (**C**, **D**) Luciferase reporter assay in HEK-293T cells showed E2F1 significantly inhibited the luciferase activity of the vector carrying promoter of FoxP3, compared with control vector. (**E**) E2F1 significantly elevated the protein expression of FoxP3 by western blotting and reduced the expression of miR-187-5p by qRT-PCR. (**F**) HBx promoted the expression of E2F1 by western blotting. The experiments were repeated at least 3 independent times.

To investigate how HBx influences FoxP3 expression, we utilized the PROMO website to predict the FoxP3 promoter (Figure 7A). In the FoxP3 promoter region, we uncovered an E2F1 binding site (Figure 7B). WT pGL3-FoxP3 (a luciferase reporter plasmid with wild type FoxP3 promoter sequence) and MUT pGL3-FoxP3 (a luciferase reporter plasmid with mutant FoxP3 promoter sequence) were constructed to verify FoxP3 promoter activity in hepatoma carcinoma cell (Figure 7C). Then we co-transfected WT pGL3-FoxP3 and MUT pGL3-FoxP3 into 293T cells with pcDNA3.1 and pcDNA3.1-E2F1. Luciferase activity was found after 36 hours transfection. The results revealed that pcDNA3.1-E2F1 transfection could considerably boost the WT pGL3-FoxP3 vector’s luciferase activity but had little effect on the MUT pGL3- FoxP3 vector’s activity (Figure 7D). We then looked at how E2F1 plasmid transfection affected the expression of FoxP3 and CDH2 in the Huh-7 cell line. When compared with the control group, E2F1 significantly increased FoxP3 protein expression (Figure 7E). The western blot findings also demonstrated that HBx increased the expression of E2F1 (Figure 7F).

## DISCUSSION

miRNAs play an important role in regulating various physiological processes, including embryonic development and tumor genesis [27–29]. However, their effects on cancer are complex and varied. By targeting ICAM1, miR-187-5p was discovered to influence bone cell development in mouse bone marrow mesenchymal stem cells [30]. According to Gong J et al., miR-187-5p promotes liver regeneration in mice via the Hippo signaling pathway [8]. Lou Y et al. discovered that MiR-187-5p could activated the Wnt/-catenin signaling pathway and regulated the development and death of acute lymphoblastic leukemia cells [31]. However, it has been discovered that miR-187-5p expression is different and can upregulate and accelerate the biological development of cancer in non-small cell lung cancer, giant cell tumor of bone, and bladder cancer [8, 9, 12]. Kurt Sartorius et al. discovered that miRNA modulation in several HBV-HCC pathways may assist the hepatitis B virus evade and manage the host immune system, as well as enhance viral replication [32]. We found that miR-187-5p was down-regulated in hepatocellular carcinoma cells in the present study and showed a decreasing trend with increasing HBx expression. Our findings suggest that increasing miR-187-5p expression can limit hepatoma carcinoma cell proliferation *in vivo*. By targeting CDH2, it influenced hepatoma carcinoma cell growth, migration, and invasion.

In HBV-related HCC, the viral protein HBx plays a major role in regulating miRNAs [33, 34]. It has been reported that HBx can promote or inhibit the expression of miRNA and thus affect the occurrence and progression of cancer [35]. Yong Gao et al. found that HBx inhibits the expression of miRNA-137 and promotes the proliferation of hepatoma carcinoma cells [33]. However, its regulation mechanism is not yet fully understood. It has been reported that HBx may regulates miRNA through methylation level [33, 34], and altering the expression of some transcription factors [36–38]. We speculate that the latter mode of regulation is also applicable to the mechanism of miR-187-5p regulation. Bioinformatic data indicated the interactions among FoxP3, miR-187-5p and E2F1, which is in line with our hypothesis that miR-187-5p may contributed to the HBx induced HCC progression, with the participation of transcription factor E2F1 and FoxP3.

Cadherin (CDH) is a member of the cell adhesion molecule family, which mediates cell-cell interactions through calcium-dependent, homologous protein interactions [39]. CDH2, encoding a 906 amino acid protein called N-cadherin, is usually found in nerve tissues, including fibroblasts. Numerous studies have shown that morphological changes in the fibroblast phenotype are usually associated with abnormal expression of N-cadherin, and causing tumor cells more motile, aggressive, and metastatic [40], which may be closely associated with tumorigenesis and metastasis. CDH2 has been confirmed to be up-regulated in breast cancer, prostate cancer, and melanoma [41]. However, it has been shown that miRNAs can inhibit CDH2 expression by binding the 3-UTR of CDH2 through sponge action [42–44].

**Figure 8:**
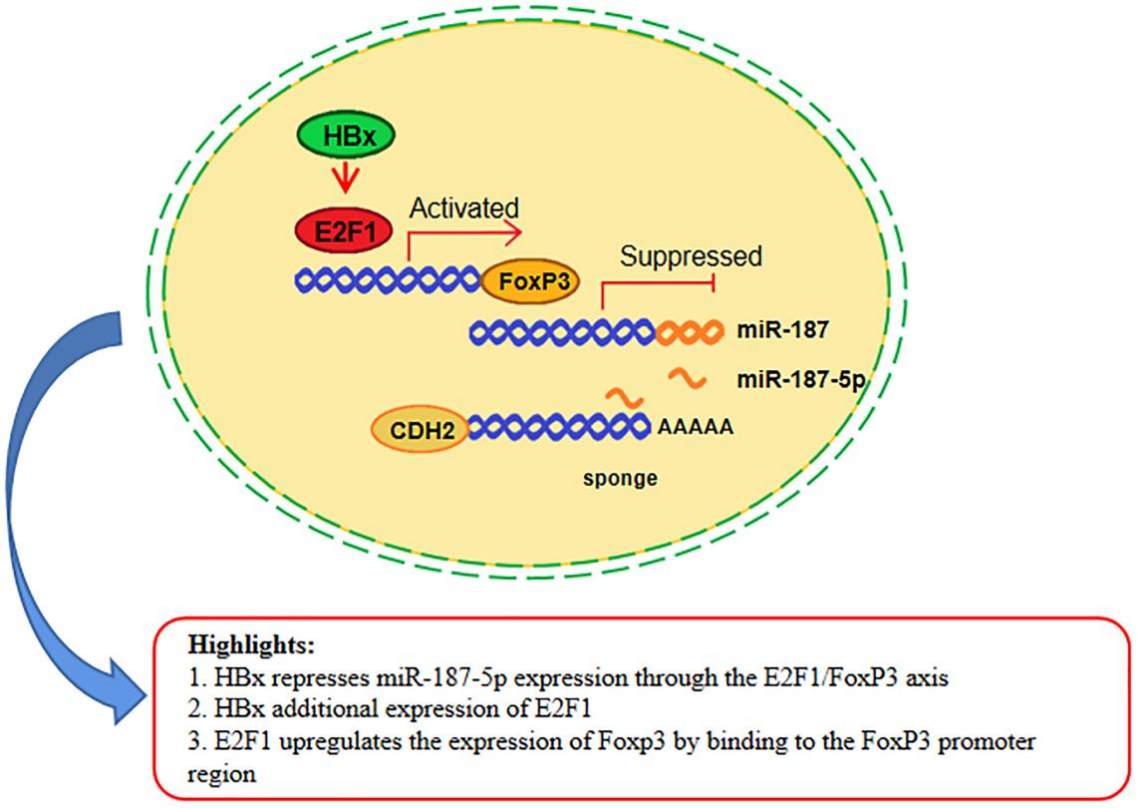
**miR-187-5p suppressed hepatoma cell proliferation, migration and invasion by sponging off CDH2 expression, which itself was inhibited by the HBx/E2F1/FoxP3 axis.** HBx promoted the expression of E2F1, which increased the expression of FoxP3 by binding to the FoxP3 promoter region. Meanwhile, the miR-187-5p promoter was suppressed by FoxP3 binding. Furthermore, miR-187-5p sponged on the 3′UTR region of CDH2, thereby inhibiting the proliferation, migration, and invasion ability of HCC cells.

Experts and scholars have been devoted to studying the mechanism of HBV on the occurrence and progression of HCC for many years. Our study found that HBx infection could promote the expression of E2F1, which were consistent with other reports [45]. Utilizing a Luciferase reporter experiment, we made the discovery that transfection of the E2F1 plasmid could enhance FoxP3 expression through binding with the 5′UTR region of FoxP3. This same region acts as a transcription factor, promoting the expression of the downstream target gene CDH2 and inhibiting miR-187-5p expression. Figure 8 summarizes our research, which shows that HBx protein can promote E2F1 expression, leading to the activation of FoxP3 expression. As a result, FoxP3 functions as a transcription factor by inhibiting the expression of miR-187-5p through its binding with the promoter of miR-187-5p. Meanwhile, miR-187-5p can bind to the 3′UTR of CDH2, inhibiting its expression and ultimately inhibiting the proliferation, migration and invasion of the hepatocellular carcinoma Huh-7 cell line (as seen in Figure 8). These findings suggest that the HBV viral protein HBx could promote HCC cell proliferation, migration, and invasion due to the inhibitory effect that E2F1 and FoxP3 have on miR-187-5P. Furthermore, miR-187-5p may play a critical role as a cancer suppressor in HCC, indicating that this study’s findings could pave the way for a novel miRNA target for the treatment of HBV-HCC. The enrichment of the miR-187-5p signaling pathway also suggests that there may be a correlation between miR-187-5p and lipid and glucose metabolism in hepatocellular carcinoma cells, which is worth further investigation.

## Supplementary Materials

Supplementary Figure 1

Supplementary Tables

## References

[1] GBD 2015 Disease and Injury Incidence and Prevalence Collaborators. Global, regional, and national incidence, prevalence, and years lived with disability for 310 diseases and injuries, 1990-2015: a systematic analysis for the Global Burden of Disease Study 2015. Lancet. 2016; 388:1545–602. 10.1016/S0140-6736(16)31678-6 27733282 PMC5055577

[2] Levrero M, Zucman-Rossi J. Mechanisms of HBV-induced hepatocellular carcinoma. J Hepatol. 2016; 64:S84–101. 10.1016/j.jhep.2016.02.021 27084040

[3] Slagle BL, Bouchard MJ. Hepatitis B Virus X and Regulation of Viral Gene Expression. Cold Spring Harb Perspect Med. 2016; 6:a021402. 10.1101/cshperspect.a021402 26747833 PMC4772081

[4] Zhang XD, Wang Y, Ye LH. Hepatitis B virus X protein accelerates the development of hepatoma. Cancer Biol Med. 2014; 11:182–90. 10.7497/j.issn.2095-3941.2014.03.004 25364579 PMC4197427

[5] Correia de Sousa M, Gjorgjieva M, Dolicka D, Sobolewski C, Foti M. Deciphering miRNAs' Action through miRNA Editing. Int J Mol Sci. 2019; 20:6249. 10.3390/ijms20246249 31835747 PMC6941098

[6] Bartel DP. MicroRNAs: genomics, biogenesis, mechanism, and function. Cell. 2004; 116:281–97. 10.1016/s0092-8674(04)00045-5 14744438

[7] Si W, Shen J, Zheng H, Fan W. The role and mechanisms of action of microRNAs in cancer drug resistance. Clin Epigenetics. 2019; 11:25. 10.1186/s13148-018-0587-8 30744689 PMC6371621

[8] Jin Y, Zhang J, Zhu H, Fan G, Zhou G. Expression profiles of miRNAs in giant cell tumor of bone showed miR-187-5p and miR-1323 can regulate biological functions through inhibiting FRS2. Cancer Med. 2020; 9:3163–73. 10.1002/cam4.2853 32154662 PMC7196053

[9] Mao M, Wu Z, Chen J. MicroRNA-187-5p suppresses cancer cell progression in non-small cell lung cancer (NSCLC) through down-regulation of CYP1B1. Biochem Biophys Res Commun. 2016; 478:649–55. 10.1016/j.bbrc.2016.08.001 27495872

[10] Matamala N, Vargas MT, González-Cámpora R, Arias JI, Menéndez P, Andrés-León E, Yanowsky K, Llaneza-Folgueras A, Miñambres R, Martínez-Delgado B, Benítez J. MicroRNA deregulation in triple negative breast cancer reveals a role of miR-498 in regulating BRCA1 expression. Oncotarget. 2016; 7:20068–79. 10.18632/oncotarget.7705 26933805 PMC4991439

[11] Ren L, Li F, Di M, Fu Y, Hui Y, Xiao G, Sun Q, Liu Y, Ren D, Du X. MicroRNA-187 regulates gastric cancer progression by targeting the tumor suppressor CRMP1. Biochem Biophys Res Commun. 2017; 482:597–603. 10.1016/j.bbrc.2016.11.079 27864146

[12] Li Z, Lin C, Zhao L, Zhou L, Pan X, Quan J, Peng X, Li W, Li H, Xu J, Xu W, Guan X, Chen Y, Lai Y. Oncogene miR-187-5p is associated with cellular proliferation, migration, invasion, apoptosis and an increased risk of recurrence in bladder cancer. Biomed Pharmacother. 2018; 105:461–9. 10.1016/j.biopha.2018.05.122 29883941

[13] Jia H, Qi H, Gong Z, Yang S, Ren J, Liu Y, Li MY, Chen GG. The expression of FOXP3 and its role in human cancers. Biochim Biophys Acta Rev Cancer. 2019; 1871:170–8. 10.1016/j.bbcan.2018.12.004 30630091

[14] Schubert LA, Jeffery E, Zhang Y, Ramsdell F, Ziegler SF. Scurfin (FOXP3) acts as a repressor of transcription and regulates T cell activation. J Biol Chem. 2001; 276:37672–9. 10.1074/jbc.M104521200 11483607

[15] Hinz S, Pagerols-Raluy L, Oberg HH, Ammerpohl O, Grüssel S, Sipos B, Grützmann R, Pilarsky C, Ungefroren H, Saeger HD, Klöppel G, Kabelitz D, Kalthoff H. Foxp3 expression in pancreatic carcinoma cells as a novel mechanism of immune evasion in cancer. Cancer Res. 2007; 67:8344–50. 10.1158/0008-5472.CAN-06-3304 17804750

[16] Ebert LM, Tan BS, Browning J, Svobodova S, Russell SE, Kirkpatrick N, Gedye C, Moss D, Ng SP, MacGregor D, Davis ID, Cebon J, Chen W. The regulatory T cell-associated transcription factor FoxP3 is expressed by tumor cells. Cancer Res. 2008; 68:3001–9. 10.1158/0008-5472.CAN-07-5664 18413770

[17] Karanikas V, Speletas M, Zamanakou M, Kalala F, Loules G, Kerenidi T, Barda AK, Gourgoulianis KI, Germenis AE. Foxp3 expression in human cancer cells. J Transl Med. 2008; 6:19. 10.1186/1479-5876-6-19 18430198 PMC2386447

[18] Grover P, Goel PN, Piccirillo CA, Greene MI. FOXP3 and Tip60 Structural Interactions Relevant to IPEX Development Lead to Potential Therapeutics to Increase FOXP3 Dependent Suppressor T Cell Functions. Front Pediatr. 2021; 9:607292. 10.3389/fped.2021.607292 33614551 PMC7888439

[19] Wang WH, Jiang CL, Yan W, Zhang YH, Yang JT, Zhang C, Yan B, Zhang W, Han W, Wang JZ, Zhang YQ. FOXP3 expression and clinical characteristics of hepatocellular carcinoma. World J Gastroenterol. 2010; 16:5502–9. 10.3748/wjg.v16.i43.5502 21086571 PMC2988246

[20] Slansky JE, Farnham PJ. Introduction to the E2F family: protein structure and gene regulation. Curr Top Microbiol Immunol. 1996; 208:1–30. 10.1007/978-3-642-79910-5_1 8575210

[21] Dyson N. The regulation of E2F by pRB-family proteins. Genes Dev. 1998; 12:2245–62. 10.1101/gad.12.15.2245 9694791

[22] Polager S, Ginsberg D. E2F - at the crossroads of life and death. Trends Cell Biol. 2008; 18:528–35. 10.1016/j.tcb.2008.08.003 18805009

[23] Kent LN, Leone G. The broken cycle: E2F dysfunction in cancer. Nat Rev Cancer. 2019; 19:326–38. 10.1038/s41568-019-0143-7 31053804

[24] Wang L, Xu C, Sun D, Deng Y, Wei X, Zhou J. Toward Understanding on the Regulatory Network of HBx-induced microRNA-187-5p in Hepatocellular Carcinoma: A Study based on Bioinformatics Analysis. ICBBT 2021: 2021 13th International Conference on Bioinformatics and Biomedical Technology. 2021; 180–6. 10.1145/3473258.3473285

[25] Chehrehasa F, Meedeniya AC, Dwyer P, Abrahamsen G, Mackay-Sim A. EdU, a new thymidine analogue for labelling proliferating cells in the nervous system. J Neurosci Methods. 2009; 177:122–30. 10.1016/j.jneumeth.2008.10.006 18996411

[26] Liao XH, Wang N, Liu LY, Zheng L, Xing WJ, Zhao DW, Sun XG, Hu P, Dong J, Zhang TC. MRTF-A and STAT3 synergistically promote breast cancer cell migration. Cell Signal. 2014; 26:2370–80. 10.1016/j.cellsig.2014.07.023 25038455

[27] Selbach M, Schwanhäusser B, Thierfelder N, Fang Z, Khanin R, Rajewsky N. Widespread changes in protein synthesis induced by microRNAs. Nature. 2008; 455:58–63. 10.1038/nature07228 18668040

[28] Wang P, Li Z, Liu H, Zhou D, Fu A, Zhang E. MicroRNA-126 increases chemosensitivity in drug-resistant gastric cancer cells by targeting EZH2. Biochem Biophys Res Commun. 2016; 479:91–6. 10.1016/j.bbrc.2016.09.040 27622325

[29] Yoo JO, Kwak SY, An HJ, Bae IH, Park MJ, Han YH. miR-181b-3p promotes epithelial-mesenchymal transition in breast cancer cells through Snail stabilization by directly targeting YWHAG. Biochim Biophys Acta. 2016; 1863:1601–11. 10.1016/j.bbamcr.2016.04.016 27102539

[30] Sun Y, Wang X, Chen G, Song C, Ma X, Fu Y, Feng C, Yan J. miRNA-187-5p Regulates Osteoblastic Differentiation of Bone Marrow Mesenchymal Stem Cells in Mice by Targeting ICAM1. Biomed Res Int. 2020; 2020:6139469. 10.1155/2020/6139469 33381563 PMC7748902

[31] Lou Y, Liu L, Zhan L, Wang X, Fan H. miR-187-5p Regulates Cell Growth and Apoptosis in Acute Lymphoblastic Leukemia via DKK2. Oncol Res. 2016; 24:89–97. 10.3727/096504016X14597766487753 27296949 PMC7838722

[32] Sartorius K, Makarova J, Sartorius B, An P, Winkler C, Chuturgoon A, Kramvis A. The Regulatory Role of MicroRNA in Hepatitis-B Virus-Associated Hepatocellular Carcinoma (HBV-HCC) Pathogenesis. Cells. 2019; 8:1504. 10.3390/cells8121504 31771261 PMC6953055

[33] Gao Y, Gu J, Wang Y, Fu D, Zhang W, Zheng G, Wang X. Hepatitis B virus X protein boosts hepatocellular carcinoma progression by downregulating microRNA-137. Pathol Res Pract. 2020; 216:152981. 10.1016/j.prp.2020.152981 32527447

[34] Zhang T, Zhang J, Cui M, Liu F, You X, Du Y, Gao Y, Zhang S, Lu Z, Ye L, Zhang X. Hepatitis B virus X protein inhibits tumor suppressor miR-205 through inducing hypermethylation of miR-205 promoter to enhance carcinogenesis. Neoplasia. 2013; 15:1282–91. 10.1593/neo.131362 24339740 PMC3858896

[35] Yang Z, Li J, Feng G, Wang Y, Yang G, Liu Y, Zhang S, Feng J, Zhang X. Hepatitis B virus X protein enhances hepatocarcinogenesis by depressing the targeting of *NUSAP1* mRNA by *miR-18b*. Cancer Biol Med. 2019; 16:276–87. 10.20892/j.issn.2095-3941.2018.0283 31516748 PMC6713641

[36] Benn J, Su F, Doria M, Schneider RJ. Hepatitis B virus HBx protein induces transcription factor AP-1 by activation of extracellular signal-regulated and c-Jun N-terminal mitogen-activated protein kinases. J Virol. 1996; 70:4978–85. 10.1128/JVI.70.8.4978-4985.1996 8764004 PMC190451

[37] Wang J, Li N, Huang ZB, Fu S, Yu SM, Fu YM, Zhou PC, Chen RC, Zhou RR, Huang Y, Hu XW, Fan XG. HBx regulates transcription factor PAX8 stabilization to promote the progression of hepatocellular carcinoma. Oncogene. 2019; 38:6696–710. 10.1038/s41388-019-0907-2 31391550

[38] Waris G, Huh KW, Siddiqui A. Mitochondrially associated hepatitis B virus X protein constitutively activates transcription factors STAT-3 and NF-kappa B via oxidative stress. Mol Cell Biol. 2001; 21:7721–30. 10.1128/MCB.21.22.7721-7730.2001 11604508 PMC99943

[39] Coulon S, Heindryckx F, Geerts A, Van Steenkiste C, Colle I, Van Vlierberghe H. Angiogenesis in chronic liver disease and its complications. Liver Int. 2011; 31:146–62. 10.1111/j.1478-3231.2010.02369.x 21073649

[40] Truong Quang BA, Mani M, Markova O, Lecuit T, Lenne PF. Principles of E-cadherin supramolecular organization in vivo. Curr Biol. 2013; 23:2197–207. 10.1016/j.cub.2013.09.015 24184100

[41] Liu YA, Liang BY, Guan Y, You J, Zhu L, Chen XP, Huang ZY. Loss of N-cadherin is associated with loss of E-cadherin expression and poor outcomes of liver resection in hepatocellular carcinoma. J Surg Res. 2015; 194:167–76. 10.1016/j.jss.2014.09.031 25438959

[42] Li M, Zhang H, Kong Q. Long non-coding RNA IGF2-AS promotes trophoblast cell proliferation, migration, and invasion by regulating miR-520g/N-cadherin axis. J Obstet Gynaecol Res. 2021; 47:3047–59. 10.1111/jog.14886 34109707

[43] Yao Y, Chen S, Lu N, Yin Y, Liu Z. LncRNA JPX overexpressed in oral squamous cell carcinoma drives malignancy via miR-944/CDH2 axis. Oral Dis. 2021; 27:924–33. 10.1111/odi.13626 32881231

[44] Zhang D, Yang XJ, Luo QD, Fu DL, Li ZL, Zhang P, Chong T. Down-Regulation of Circular RNA_000926 Attenuates Renal Cell Carcinoma Progression through miRNA-411-Dependent CDH2 Inhibition. Am J Pathol. 2019; 189:2469–86. 10.1016/j.ajpath.2019.06.016 31476285

[45] Ghosh A, Ghosh S, Dasgupta D, Ghosh A, Datta S, Sikdar N, Datta S, Chowdhury A, Banerjee S. Hepatitis B Virus X Protein Upregulates hELG1/ ATAD5 Expression through E2F1 in Hepatocellular Carcinoma. Int J Biol Sci. 2016; 12:30–41. 10.7150/ijbs.12310 26722215 PMC4679396

