# Supplementary material for: HBx promotes hepatocellular carcinoma progression by repressing the transcription level of miR-187-5p
Source: Aging (Albany NY). 2023 Aug 1;15(15):7533–50. doi: 10.18632/aging.204921 (PMC10457053; doi:10.18632/aging.204921)
Supplement: Supplementary Figure 1 [file aging-15-204921-s001.pdf]

SUPPLEMENTARY FIGURE

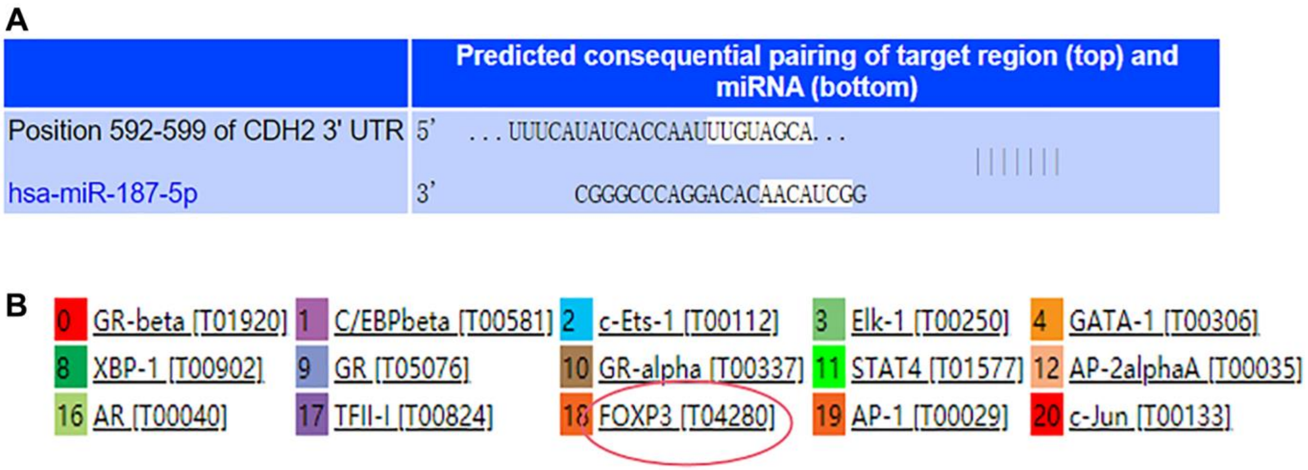

Supplementary Figure 1. Prediction of binding sites using websites. (**A**) miR-187-5p bound to 3'-UTR of CDH2 found by TargetScanHuman Website. (**B**) Used PROMO website to predict the promoter of miR-187-5p.
