# Supplementary material for: HBx promotes hepatocellular carcinoma progression by repressing the transcription level of miR-187-5p
Source: Aging (Albany NY). 2023 Aug 1;15(15):7533–50. doi: 10.18632/aging.204921 (PMC10457053; doi:10.18632/aging.204921)
Supplement: Supplementary Tables [file aging-15-204921-s002.pdf]

SUPPLEMENTARY TABLES

Supplementary Table 1. Details of plasmid construction primer sequences.

Gene name	Primer sequences (5′–3′)
E2F1(F)	CATCCCAGGAGGTCACTTCTG
E2F1(R)	GACAACAGCGGTTCTTGCTC
FoxP3(F)	ATGCCCAACCCCAGGCCTGGCAA
FoxP3(R)	TCAGGGGCCAGGTGTAGGGTTGGA
WT-pmirGLO-CDH2(F)	TGTTTAAACGAGCTCGCTAGCACTTCAGGGTGAACTTGGT
WT-pmirGLO-CDH2(R)	GACTCTAGACTCGAGGCTAGCCCAGATCCAAAATTAGCA
MUT-pmirGLO-CDH2(F)	CACCAATTTGGCACAAAATTGAATTTTTTCATAAAC
MUT-pmirGLO-CDH2(R)	TTTGTGCCAAATTGGTGATATGAAAACTCCCT
WT-pGL3-miR-187-5p(F)	CGAGCTCTTACGCGTGCTAGCTCCCTCCCTCTAAATTGTTC
WT-pGL3-miR-187-5p(R)	AGATCTCGAGCCCGGGCTAGCTGCGGACCTGCGTCCCT
MUT miR-187-5p(F1)	AAGAAACCATCCGTAAGTAATAAATCCTTCAGAGGCC
MUT miR-187-5p(R1)	GGCTGCAGACAAGCCACTACAAGCCACTAATTACAGTTTA
MUT miR-187-5p(F2)	ATTACTTATGGCTGGTTTCTTTTTATTCAAA
MUT miR-187-5p(R1)	AGTGGCTTGTCTGCAGCCATAATTCATTGTTTAAAGGA
WT-pGL3-FoxP3(F)	CGAGCTCTTACGCGTGCTAGC CAGCCCCCTTGTAGACCTTGA
WT-pGL3-FoxP3(R)	AGATCTCGAGCCCGGGCTAGCGGCTTGGTGAAGTGGACTGA

Supplementary Table 2. Details of qRT-PCR primer sequence.

Gene name	Primer sequences (5′–3′)
E2F1(F)	GTGGCCCGGATGTGAGAAG
E2F1(R)	GGAGCCCTTGTCGGATGATG
FoxP3(F)	GTGGCCCGGATGTGAGAAG
FoxP3(R)	GGAGCCCTTGTCGGATGATG
CDH2(F)	TGCGGTACAGTGTAACTGGG
CDH2(R)	GAAACCGGGCTATCTGCTCG
HBx-flag(F)	ATGGCTGCTAGGCTGTGCT
HBx-flag(R)	TTAGGCAGAGGTGAAAAAGTTG
GAPDH(F)	CTTTGGTATCGTGGAAGGACTC
GAPDH(R)	GTAGAGGCAGGGATGATGTTCT
U6(F)	CAGCACATATACTAAAATTGGAACG
U6(R)	ACGAATTTGCGTGTCATCC
hsa-miR-187-5p(F)	TCGTGTCTTGTGTTGCAGC
hsa-miR-187-5p(R)	GTGCAGGGTCCGAGGT
